# NSUN6 and HTR7 disturbed the stability of carotid atherosclerotic plaques by regulating the immune responses of macrophages

**DOI:** 10.1515/med-2024-1072

**Published:** 2024-10-24

**Authors:** Tingyu Jin, Han Gao, Danyang Meng, Man Luo, Jin Hu

**Affiliations:** Department of Neurology, Affiliated Hospital of Jiaxing University, The First Hospital of Jiaxing, Jiaxing, Zhejiang, China; Department of Neurology, Affiliated Hospital of Jiaxing University, The First Hospital of Jiaxing, 1882 Zhonghuan South Road, Chengnan Street, Jiaxing, Zhejiang, China

**Keywords:** carotid atherosclerotic plaques, bioinformatics analysis, immune infiltration, cellular localization, macrophages

## Abstract

**Background:**

Ischemic stroke associated with atherosclerosis is globally named atherothrombotic stroke. Presently, the underlying pathogenic genes promoting carotid atherosclerotic plaques transfer from a stable to unstable state remains elusive. This study aims to find the hub genes disturbing the stability of plaques and explore the primary cells affected by these hub genes.

**Methods:**

The optimal hub genes from five datasets for unstable plaques were identified by overlapping genes derived from Boruta and LASSO algorithms. The hub genes’ expression levels in stroke patients were confirmed through RT-qPCR. Visualization of the hub genes’ expression across various cell clusters was achieved with the aid of the Seurat R package. Then, hub genes were overexpressed or knock-down by lentivirus and siRNA, respectively. The inflammatory factors in the culture medium were detected using an ELISA assay.

**Results:**

Eight genes (APOD, ASXL1, COL4A5, HTR7, INF2, NSUN6, PDSS2, and RBBP7) were identified and confirmed by RT-qPCR. The prognostic model was built upon this eight-gene composite foundation, and the area under the curve was 0.98. Based on CIBERSORT findings, unstable plaques displayed a higher macrophage proportion compared to stable ones (*P* < 0.05). These eight genes also correlated with infiltrated immune cells, especially macrophages. Then, according to single-cell RNA-seq analysis, we found that the eight hub genes mainly expressed in macrophages. The cellular localization of two hub genes (NSUN6 and HTR7) with high distinguishability was confirmed, and gene set enrichment analysis also clarified the possible biological pathways regulated by them. The findings from the *in vitro* investigation revealed that TNF-α and IL-6 were reduced in macrophages with NSUN6 overexpression or HTR7 knockdown.

**Conclusion:**

Eight hub genes, especially NSUN6 and HTR7, were found to promote the progression of plaques by regulating the immune responses of macrophages.

## 1 Introduction

Globally, stroke serves as a primary contributor to enduring disability, cognitive decline, and mortality. Eighty percent of them are ischemic [1], and approximately 18–25% of them are attributable to thromboembolism caused by carotid atherosclerosis [2]. In accordance with the categorization of acute ischemic stroke subtypes, ischemic stroke linked to atherosclerosis is universally referred to as atherothrombotic stroke [3]. The formation of atherosclerotic plaques is a slow and progressive process, and plaques develop from asymptomatic to a late stage during which thrombi are formed, triggering ischemic cerebrovascular events [4]. Atherosclerosis represents a persistent inflammatory process, initiated by lipoprotein retention within the subendothelial space of arterial walls and subsequent acquisition of inflammatory characteristics [5]. Lipoprotein retention induces the expression of some inflammation-related factors, which attracts circulating leukocytes to the site of the lesion [6]. Within plaques, infiltrating monocytes differentiate into macrophages, which react to inflammation and participate in the formation of the fibrous cap. If the opposing reaction fails to combat inflammation, stable atherosclerotic plaques may advance and transform into unstable ones [7]. Therefore, the inflammatory cells and immunological signaling pathways hold a pivotal function in the progression of plaque formation [8]. Past research has indicated that multiple varieties of immune cells and inflammatory cytokines are involved in this chronic progress [9–11]. However, it is still unclear about the change of immune cell infiltration in plaques when they transfer from a stable to an unstable state. The molecular mechanisms promoting the development of plaques are also unclear.

In recent times, alongside the swift advancement of high-throughput techniques, RNA-sequence analysis, and gene microarray have become effective methods for exploring differentially expressed genes (DEGs) in various diseases. Multiple studies have identified several key genes and related pathways for atherosclerotic plaques with these technologies [12–14]. However, the cellular localization and specific mechanisms of these identified genes and uncovered key genes have not been clarified. Since carotid atherosclerosis is closely related to immune reaction, an exhaustive bioinformatics examination focusing on immune-related regulation ought to be conducted.

In the present study, four-gene expression series from microarray were enrolled to identify the hub genes that promote the plaques transfer from stable to unstable state, and one single-cell RNA sequencing gene expression information was included for assist to clarify the specific cellular localization of these hub genes. The distinguishing ability of these hub genes was evaluated by a computational method named LGBMMDA, and the alteration in immune cell infiltration within plaques was examined utilizing CIBERSORT. Then, analysis of single-cell RNA-seq (scRNA-seq) showed the cellular localization of these hub genes. This study will not only identify key genes with potential as prospective biomarkers for evaluating the vulnerability of plaques but also explore the underlying mechanisms of plague’s progression, providing new targets and strategies for plaques therapy.

## 2 Materials and methods

### 2.1 Datasets acquisition

GSE41571, GSE111782, GSE118481, and GSE12828, which contained reliable samples sources from *Homo sapiens*, were retrieved from the GEO database utilizing the R software’s GEO query package (version 4.1.1). GSE41571 contained microarray data from five unstable and six stable plaques; GSE111782 contained nine unstable and nine stable plaques; GSE118481 contained ten unstable and six stable plaques, and GSE12828 contained six unstable plaques (Table 1). The classification of stable and unstable plaques is based on histological criteria or clinical (symptomatic or asymptomatic) [15].

**Table 1:** Characteristics of gene microarray of this study

Reference	GEO	Platform	Stable plaques	Unstable plaques
Lee et al.	GSE41571	GPL570	5	6
Caparosa et al.	GSE111782	GPL571	9	9
Chai et al.	GSE118481	GPL10558	6	10
Hägg et al.	GSE12828	GPL571	0	6

### 2.2 Data pre-processing

After downloading series matrix files of GSE41571, GSE111782, GSE118481, and GSE12828 from GEO, probes were annotated with the annotation profile, and non-matched probes were removed. Subsequently, the four datasets were combined utilizing the sva R package [16]. Principal component analysis (PCA) was utilized to test and ensure the success of batch removal.

### 2.3 Analysis of DEGs

Utilizing the limma R package, we investigated the DEGs between stable and unstable plaques. DEGs with a statistically significant cut-off were characterized by *P* < 0.05 and log(FC) > 0.5. A heatmap was employed to visualize the DEGs using the pheatmap R package. Up and down-regulated genes were obtained independently and utilized for further analysis.

The pathway enrichment of DEGs in the Kyoto Encyclopedia of Genes and Genomes (KEGG) [14] pathways were functionally analyzed by the Cluster Profiler R package [17]. A threshold of *P* < 0.05 was established. The visualization of the results from KEGG was conducted by the ggplot2 R package.

### 2.4 Identification of hub genes

The relevance of features was compared to that of random probes using the Boruta algorithm. Then the LASSO algorithm was used to reduce the dimensionality of these data [18]. The DEGs between stable and unstable plaques were acquired for feature selection, and hub genes for unstable plaques were identified with the Boruta and LASSO algorithms. Additionally, by overlapping genes obtained from the two algorithms, the ideal hub genes associated with unstable plaques were identified. The prediction model was conducted by a computational method named LGBMMDA.

The evaluation of the discovered hub genes’ diagnostic potential was carried out through the application of receiver operating characteristic curve (ROC), with the area under the curve (AUC) acting as the primary performance metric. To determine if these hub genes could distinguish between stable and unstable plaque samples, PCA was employed.

### 2.5 RT-qPCR validation of hub genes

Serum samples of eight patients with stable carotid atherosclerotic plaques and ten patients with unstable plaques were collected for RT-qPCR verification to confirm the hub genes’ expression. Total RNA extraction was carried out. From the total RNA, RNA was reversed to cDNA, followed by RT-qPCR. Table 2 displays the primer sequences employed in this study.

**Table 2:** Primers for mRNA real-time polymerase chain reaction

Gene	Primer (5′–3′)
PDSS2	F: GCCACGTTATCTTGGAGCCT
	R: GTCATGTACAAGCCCCCTGG
INF2	F: GGAGCCAGGAAGGCCTCA
	R: GGCACGGAGTTTTGGTTTCC
HTR7	F: GGGACCTGAGGACCACCTAT
	R: CAGTAGTCAGCATTTTGTAGCAC
RBBP7	F: GATGGGGATTGGAGAGACCA
	R: AATCTTTTCCTTCAGGTTTAGTCAC
NSUN6	F: GAGGAGCCCATGTCTATGCC
	R: ATGCCCATGCCTTTCAGTTC
ASXL1	F: CCTTTTCACGCTCAAGGTGTG
	R: CCACTCCCAAGCTTACAGCA
COL4A5	F: TGGGCCCCAAGGTCCTC
	R: GGTCCTTTCATGCCTGGGAA
APOD	F: TTCATCTTGGGAAGTGCCCC
	R: TGCCGATGGCATAAACCAGG
GAPDH	F: AATGGGCAGCCGTTAGGAAA
	R: GCCCAATACGACCAAATCAGAG

### 2.6 Immune infiltration by CIBERSORT analysis

The CIBERSORT algorithm was utilized to investigate immune cell infiltration in carotid plaques [19,20]. The fraction of 22 sorted immune cell subtypes was assessed to identify the relationship between every cellular type and carotid plaque stability. Only data with a CIBERSORT *P* value <0.05 were retained for further analysis. Results obtained from the CIBERSORT analysis were visualized by the vioplot and ggplot2.

### 2.7 scRNA-seq datasets and data pre-processing

scRNA-seq gene expression data of calcified atherosclerotic core (AC) plaques and patient-matched proximal adjacent (PA) were included from the GEO database (GSE159677), and the barcode information and expression matrix were extracted. The scRNA-seq data were subjected to quality control, analysis, and exploration using Seurat, a tool commonly employed in single-cell genomics [21].

### 2.8 Detection of highly variable genes and cell clustering analysis

To remove any dimensional relationship between variable genes, the log-normalize method was used for data normalization. The FindVariableFeature function was utilized to calculate the standard variance of each gene across all cells, resulting in the mean variance being set as the standard variance. Highly variable genes were identified using a cut-off value of 1.

In this study, PCA was performed based on highly variable genes. The cell clustering was visualized through the RunUMAP function using the selected PCs as input. The FindAllMarkers function was utilized to identify genes with enriched expression in each cell cluster in the dataset. These representative genes, along with previously established markers for cell types typically found in human atherosclerotic plaques, were utilized to determine the cell identity of each cluster. The FindNeighbors and FindClusters functions were employed for the second-level clustering, with a resolution of 0.1 used for the FindClusters function.

### 2.9 Cellular localization and gene set enrichment analysis (GSEA) of hub genes

Hub genes that can discriminate stable plaque samples from unstable plaque samples were imported. The expression of the hub genes across various cell clusters was depicted. Then the Seurat package was used to search for the cell identity of the excavated genes. According to the accuracy of these hub genes in distinguishing different plaque statues, the hub genes with high AUC values were selected. We inferred the cellular localization using the FeaturePlot function. Guilt by association and GSEA were used to predict hub genes’ functions [22]. The Cluster-Profiler and ggplot2 R packages were utilized for conducting the GSEA.

### 2.10 Cell culture

Mouse mononuclear macrophage cells Raw 264.7 (CSTR:19375.09.3101MOUSCSP5036) were acquired from the National Collection of Authenticated Cell Cultures. Raw 264.7 cells were maintained in high-glucose DMEM, supplemented with 10% fetal bovine serum and 1% streptomycin/penicillin at 37°C with a 5% concentration of carbon dioxide.

### 2.11 5-Hydroxytryptamine receptor 7 (HTR7) siRNA transfection/NSUN6 overexpression

Genomeditech Co., Ltd (Shanghai, China) provided the HTR7 siRNA. To achieve overexpression of NSUN6, lentivirus virus transfection was utilized to integrate the NSUN6 genome (Genomeditech, Shanghai, China) into the genome of RAW 264.7 cells.

### 2.12 ELISA

Raw 264.7 cells were seeded into six-well plates at a density of 1 × 10^−6^ cells per well and incubated for 24 h to allow for proper cell adhesion. Afterward, cells were stimulated with 100 ng/mL lipopolysaccharide (LPS) for the desired treatment period. Post-treatment, the cells were carefully collected from each well and transferred into sterile Eppendorf tubes for further processing. The harvested cells were resuspended in phosphate-buffered saline to prepare the cell suspensions. To release intracellular components, the suspensions were subjected to a series of freeze–thaw cycles, designed to lyse the cells completely. Following cell lysis, the samples were centrifuged at 3,000 rpm for 20 min to separate the cellular debris from the supernatant. The supernatant, containing the target cytokines, was collected and used for quantifying levels of TNF-α and IL-10. ELISA assays were performed using TNF-α (RK05032, Abclonal) and IL-10 (RK00008, Abclonal) enzyme-linked immunosorbent assay kits, according to the manufacturer’s instructions.

### 2.13 Statistical analysis

The Wilcoxon test was utilized to determine if a statistically significant difference existed among the groups. All *P* values were considered two-sided, and a *P* < 0.05 was considered statistically significant. All statistical analyses and result visualization were conducted using R software (v4.1.1, packages “pheatmap,” “sva,” “limma,” “ggplot2,” “Boruta,” “glmnet,” “dplyr,” “e1071,” “Seurat,” “patchwork,” “readxl,” and “clusterProfiler”).


**Ethical approval:** The ethical review board of the First Hospital of Jiaxing, Zhejiang, China, approved the clinical study and animal experiment protocol and strictly followed its guidelines (Ethical approval number: 2023-LP-014).
**Consent to participate:** Informed consent was obtained from the patients or their legal representatives.

## 3 Results

### 3.1 A total of 338 DEGs were identified between stable and unstable plaques

**Figure 1:**
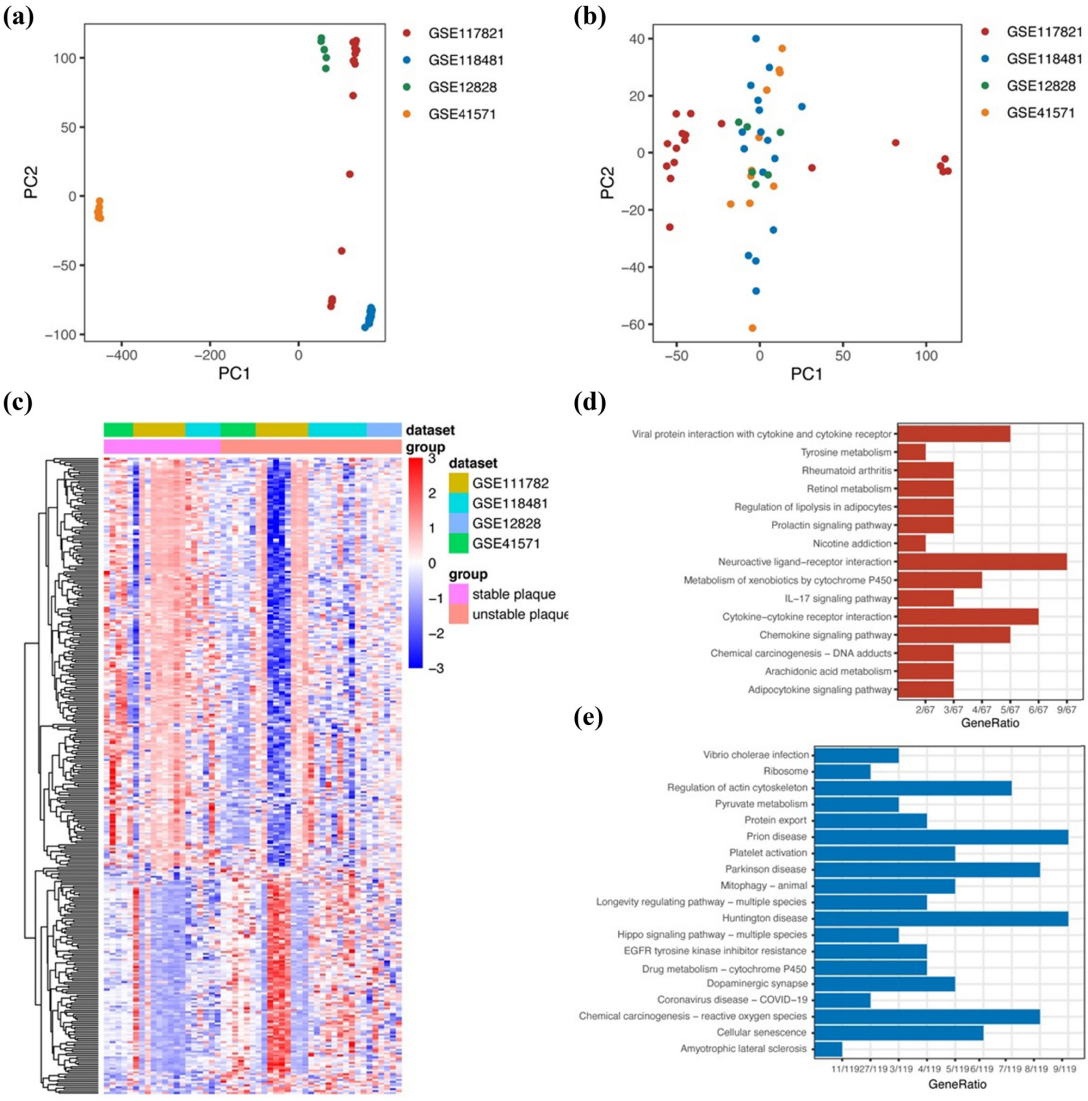
DEGs in unstable carotid atherosclerotic plaques. (a) PCA before batch removal. (b) PCA after batch removal. (c) Heatmap shows DEGs from a merged raw dataset (GSE41571, GSE111782, GSE118481, and GSE12828). (d) KEGG analysis of the upregulated genes. (e) KEGG analysis of the downregulated genes.

The expression matrices from four raw datasets (GSE41571, GSE111782, GSE118481, and GSE12828) were merged and then pre-processed for eliminating batch effect using the sva package. The result of PCA indicated that the batch effect among the different datasets was eliminated successfully (Figure 1a and b). Using the limma R package, we identified 338 DEGs between stable and unstable plaques (Figure 1c). As shown in Figure 1d, analysis of the KEGG pathways demonstrated that the elevated genes exhibited notable enrichment in a range of diverse processes. KEGG analysis results of downregulated genes included pyruvate metabolism, Hippo signaling pathway, platelet activation, etc. (Figure 1e).

### 3.2 Eight hub genes were identified to distinguish the unstable plaque from the stable plaque

**Figure 2:**
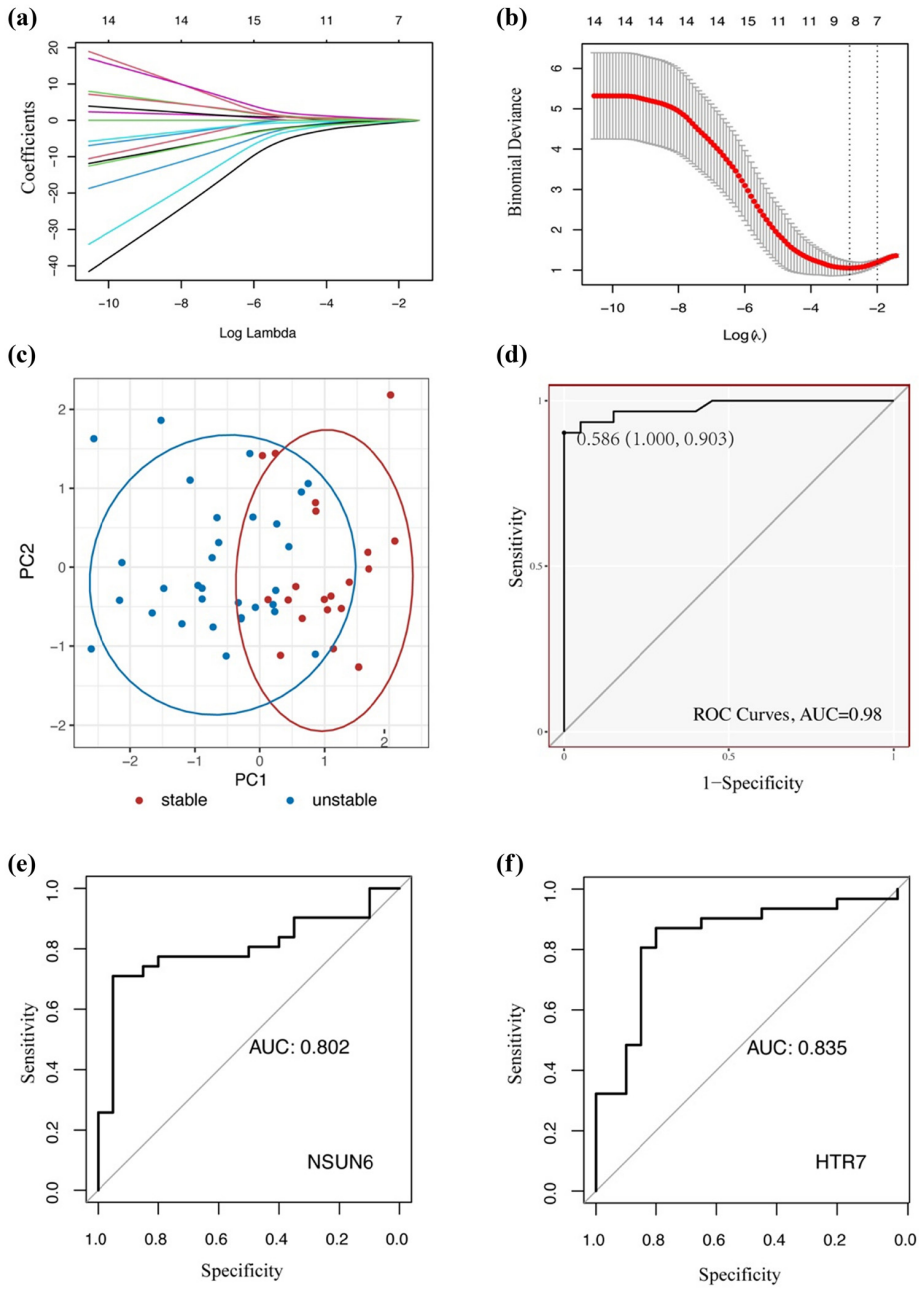
Identification of hub genes. (a) and (b) Visualization of parameters of LASSO algorithm. (c) PCA indicates that hub genes can distinguish the unstable plaque from stable plaque. (d) Prediction model based on the eight-gene combination. (e) ROC curve of NSUN6. (f) ROC curve of HTR7.

To explore the hub genes for different states of plaque, LASSO and Boruta algorithms were performed. Eight genes were identified after feature selection and dimensional reduction with these two algorithms (Figure 2a and b). The eight hub genes included apolipoprotein D (APOD), additional sex combs like 1 (ASXL1), collagen type IV alpha 5 chain (COL4A5), HTR7, inverted formin 2 (INF2), NOP2/Sun RNA methyltransferase family member 6 (NSUN6), decaprenyl diphosphate synthase subunit 2 (PDSS2), and retinoblastoma binding protein 7 (RBBP7) (Table 3). The result of PCA indicated that these eight hub genes could effectively distinguish the unstable plaque from the stable plaque (Figure 2c). The prediction model was constructed based on this eight-gene combination, and the AUC of the model was 0.98 with a specificity of 1 and sensitivity of 0.903 (Figure 2d). Next, ROC was calculated to evaluate the accuracy of each hub gene in distinguishing stable and unstable plaques (Figure S1). The up and down-regulated hub genes with the highest value of AUC were NSUN6 (AUC = 0.802, Figure 2e) and HTR7 (AUC = 0.835, Figure 2f), respectively.

**Table 3:** Eight hub genes

Name	Full name	logFC	*P* value
PDSS2	Decaprenyl diphosphate synthase subunit 2	−0.6152791	0.00028421
INF2	Inverted formin 2	0.52971016	0.0005246
HTR7	5-Hydroxytryptamine receptor 7	0.59772183	0.00056742
RBBP7	RB binding protein 7, chromatin remodeling factor	−0.7391175	0.00069048
NSUN6	NOP2/Sun RNA methyltransferase 6	−0.532062	0.00097979
ASXL1	ASXL transcriptional regulator 1	−0.5045419	0.00112298
COL4A5	Collagen type IV alpha 5 chain	−0.6078938	0.00434163
APOD	Apolipoprotein D	0.57578279	0.00956397

### 3.3 Expression of the eight hub genes in patients’ serum

**Figure 3:**
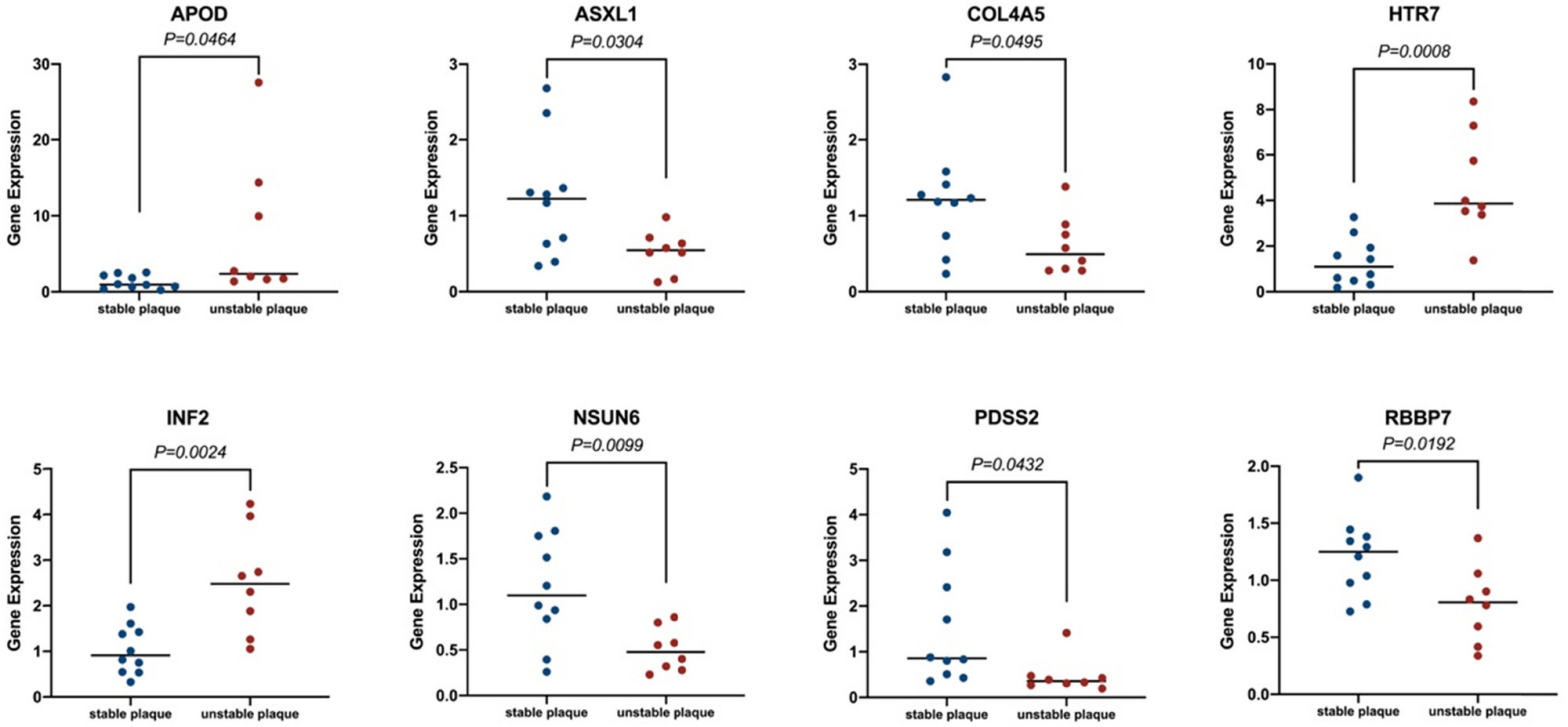
Expression levels of eight hub genes in stable and unstable carotid atherosclerotic plaques of patients’ serum.

The RT-qPCR results indicated that APOD (1.294 vs 7.674, *P* = 0.0464), HTR7 (1.319 vs 4.676, *P* = 0.0008), and INF2 (1.036 vs 2.510, *P* = 0.0024) were elevated. Conversely, the mRNA expression levels of ASXL1 (1.223 vs 0.5281, *P* = 0.0304), COL4A5 (1.208 vs 0.608, *P* = 0.0495), NSUN6 (1.188 vs 0.502, *P* = 0.0099), PDSS2 (1.516 vs 0.4748, *P* = 0.0432), and RBBP7 (1.21 vs 0.7864, *P* = 0.0192) were found to be lower. Therefore, these eight hub genes could be potential biomarkers for sensing and predicting high-risk plaques (Figure 3).

### 3.4 The eight hub genes were found to be related to the invasion of immune cells in unstable plaques

**Figure 4:**
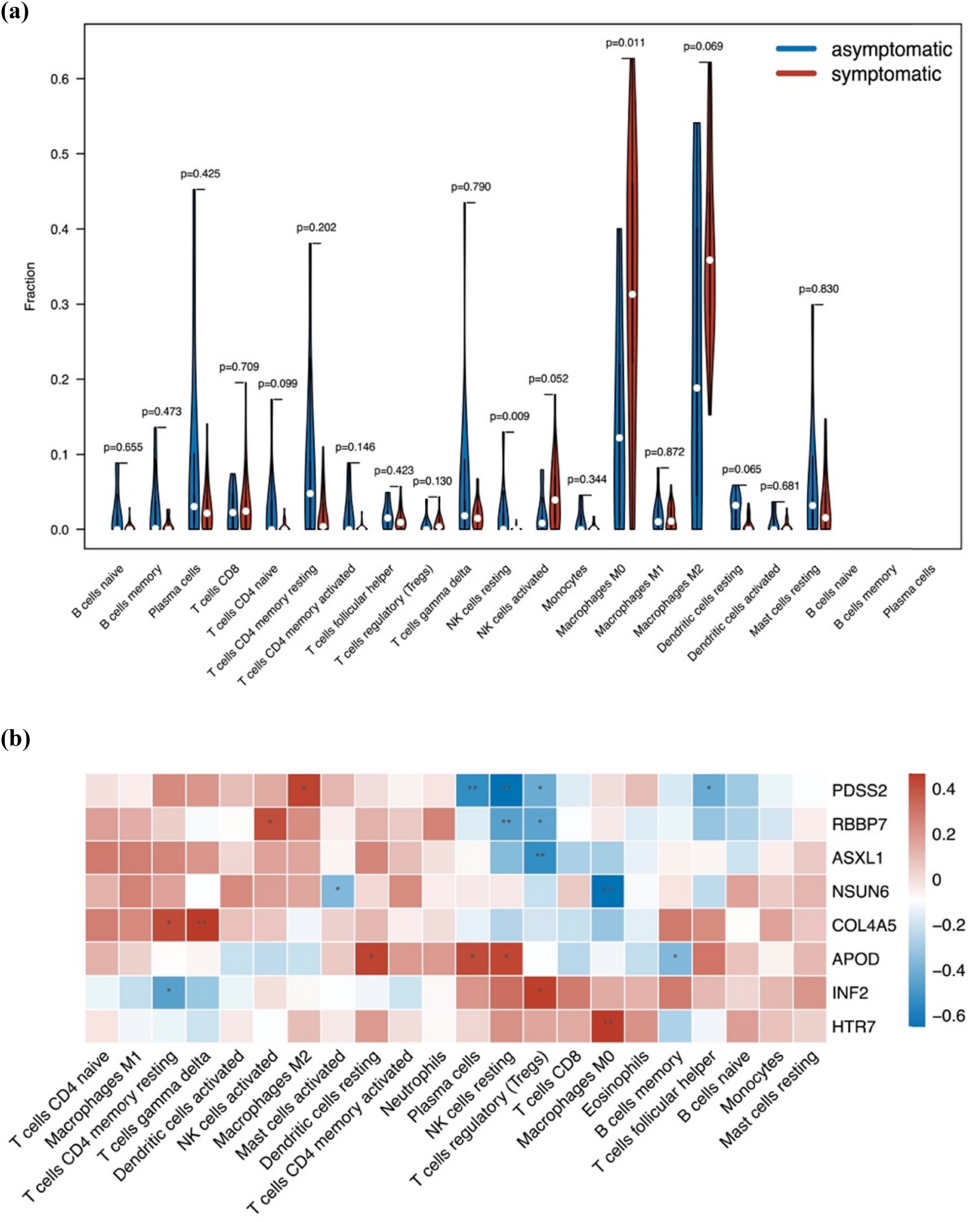
Immune infiltration between stable and unstable carotid atherosclerotic plaques. (a) Difference of immune infiltration between stable (blue) and unstable (red) carotid atherosclerotic plaques. (b) Relationship of the eight hub genes and infiltrated immune cells.

To clarify the difference between infiltrated immune cells between stable plaques and unstable plaques, we applied CIBERSORT to evaluate the immune cell abundance. Compared to stable plaques, unstable plaques were generally characterized by a higher proportion of macrophage M0 (*P* = 0.011), whereas the resting NK cells (*P* = 0.009) were lower (Figure 4a). Additionally, we investigated the correlation of the hub genes with infiltrated immune cells. Findings demonstrated a substantial association between the invasion of immune cells and the eight genes. For example, HTR7 was positively correlated with macrophages (*P* < 0.01), whereas NSUN6 was negatively correlated with macrophages (*P* < 0.01, Figure 4b).

### 3.5 Macrophages were found to increase in unstable plaques based on scRNA-seq

**Figure 5:**
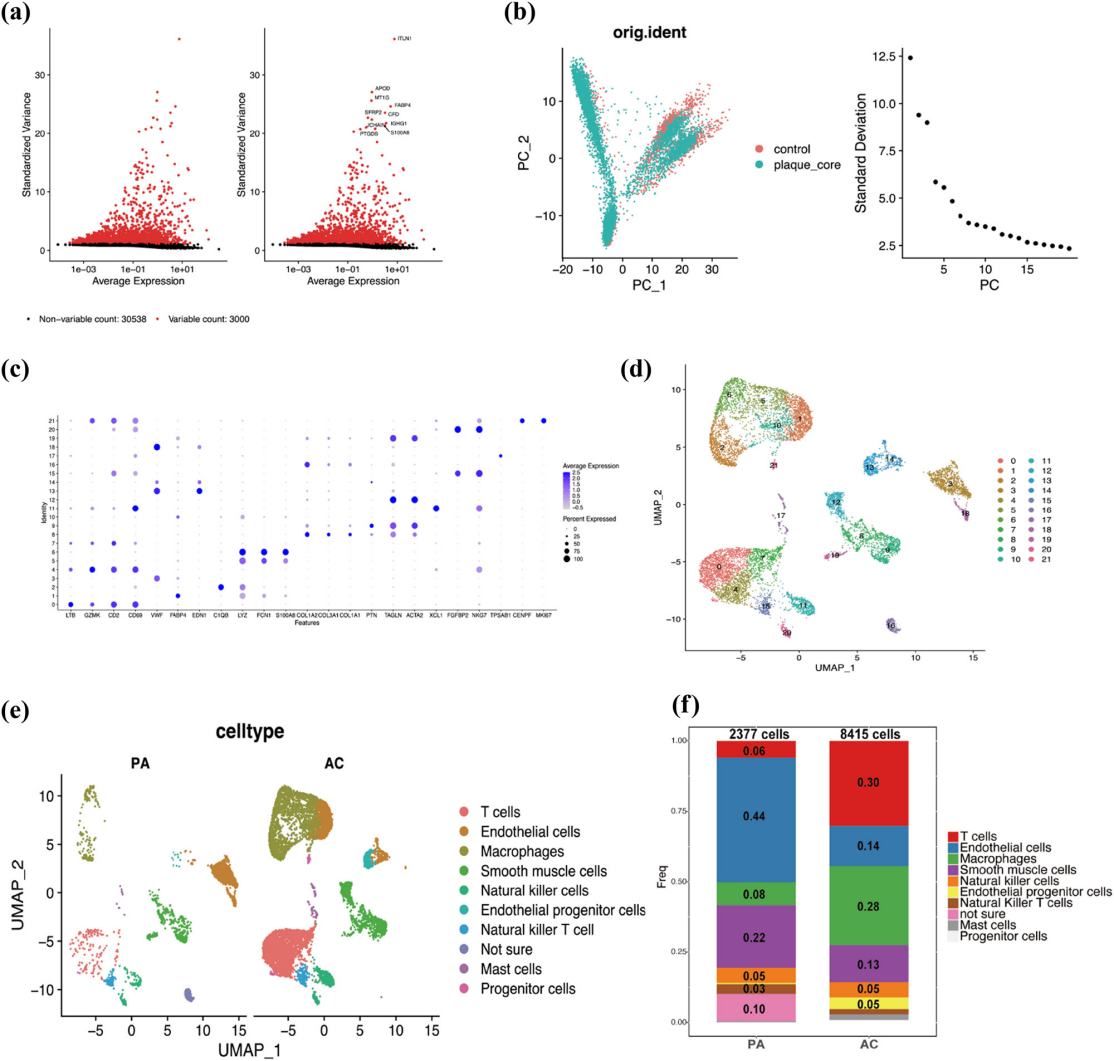
Identification of cell clusters across plaque cells based on scRNA-seq. (a) 3,000 highly variable genes. (b) Dimensional reduction and PCA. (c) Dot plot shows the cell markers. (d) UMAP shows the 22 cell clusters. (e) and (f) Difference of cell types between AC and PA groups. AC: atherosclerotic core; PA: proximal adjacent portions of carotid artery.

In this study, scRNA-seq libraries were generated using cells isolated from calcified AC plaques and PA regions of the carotid artery from the same patients. As shown in Figure S2, quality control and filtering were conducted, and cells with good-quality data were retained for further analysis. Upon calculating the average and the ratio of variance to mean for each gene, 3,000 highly variable genes were detected among the individual cells. Figure 5a displays the top ten highly variable genes, which include ITLN1, APOD, and MT1G, among others.

After dimensional reduction and PCA, a total of 20 principal components were identified based on highly variable genes. As the elbow point was obvious, we selected ten principal components for downstream analysis (Figure 5b). We divided the cells into 22 cell clusters via cluster analysis and visualized them using UMAP via the RunUMAP function (Figure 5c). Cell categories were attributed to every cluster based on the dot plot utilizing established lineage-specific marker genes (Figure 5d). We observed 10 nonimmune cell clusters and 11 leukocyte clusters. Macrophages and T cells appeared to be the most abundant populations in the AC data set, encompassing 28.1 and 30.1% of all analyzed cells. Further, it is interesting to note that macrophages have a significant difference between PA and AC, from 8.2 to 28.1% (Figure 5e and f).

### 3.6 Expression level of the eight hub genes in macrophages

**Figure 6:**
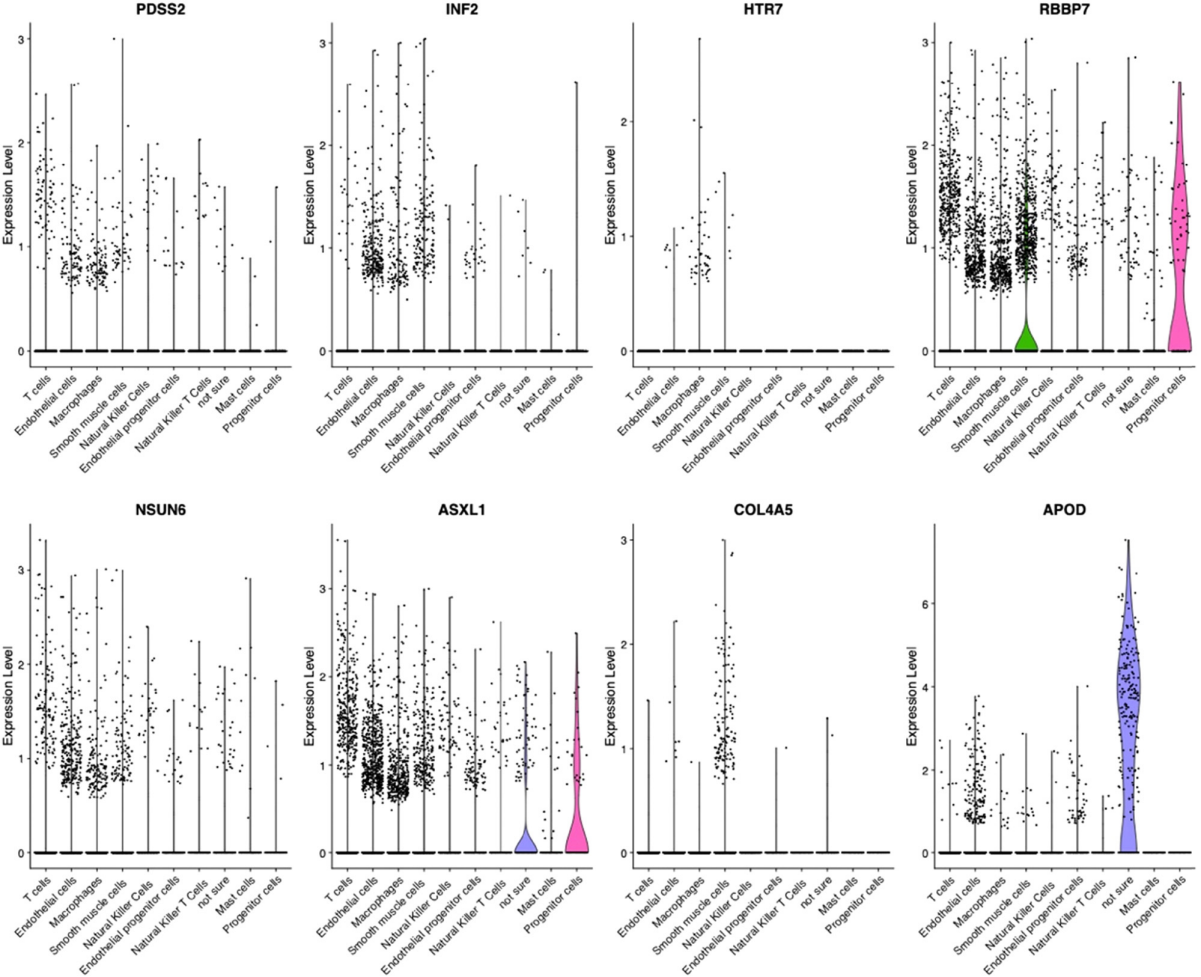
Expression level of hub genes in different cell types.

As mentioned above, we identified eight hub genes that can distinguish unstable plaques from stable plaques. Then, the scRNA-seq analysis was utilized to identify different cell types, and the hub genes were evaluated in each of these cell types. As shown in Figure 6, these eight hub genes are mainly expressed in endothelial cells, smooth muscle cells, and macrophages.

### 3.7 Two macrophage subpopulations influenced the plaque stability

**Figure 7:**
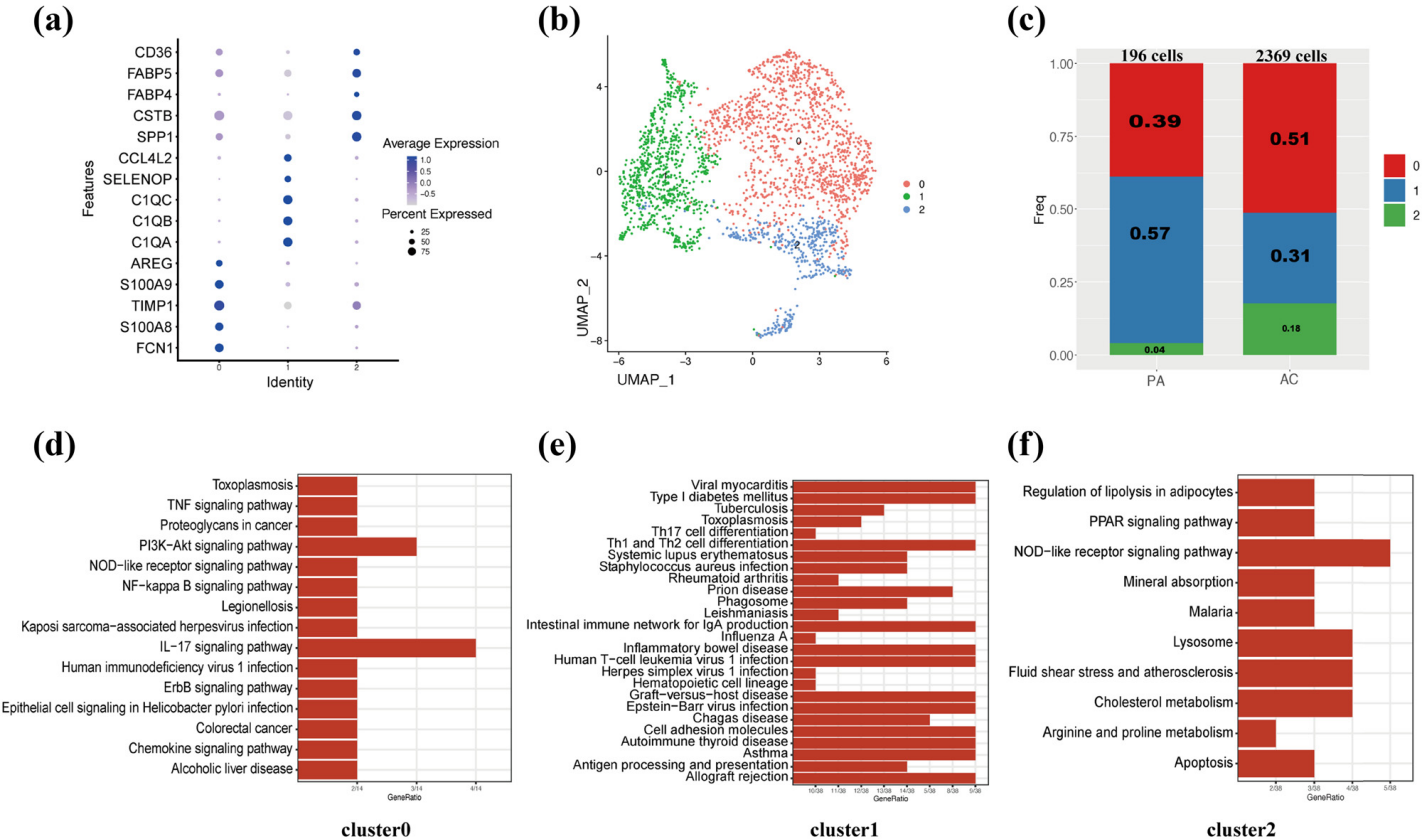
Functional and spatial signatures of macrophage subpopulations. (a) Dot plot shows the cell markers. (b) UMAP shows the three cell clusters. (c) Amount of three cell clusters. (d) KEGG analysis of cluster 0 of macrophage. (e) KEGG analysis of cluster 1 of macrophage. (f) KEGG analysis of cluster 2 of macrophage.

The “FindClusters” algorithm was employed to identify three distinct subpopulations of macrophages (Figure 7a and b). The purpose of this was to explore if specific functions could be attributed to each subpopulation. A significant difference in the percentage of these three subpopulations was observed between PA and AC groups (Figure 7c). KEGG analyses of these three clusters indicated that cluster 0 was related to pro-inflammatory pathways and functions, while cluster 2 was related to lipid metabolism such as regulation of lipolysis in adipocytes and cholesterol metabolism (Figure 7d–f). These results indicated that cluster 0 and cluster 2 of macrophages may affect the development of plaques.

### 3.8 Cellular localization and GSEA of NSUN6 and HTR7 in macrophages

**Figure 8:**
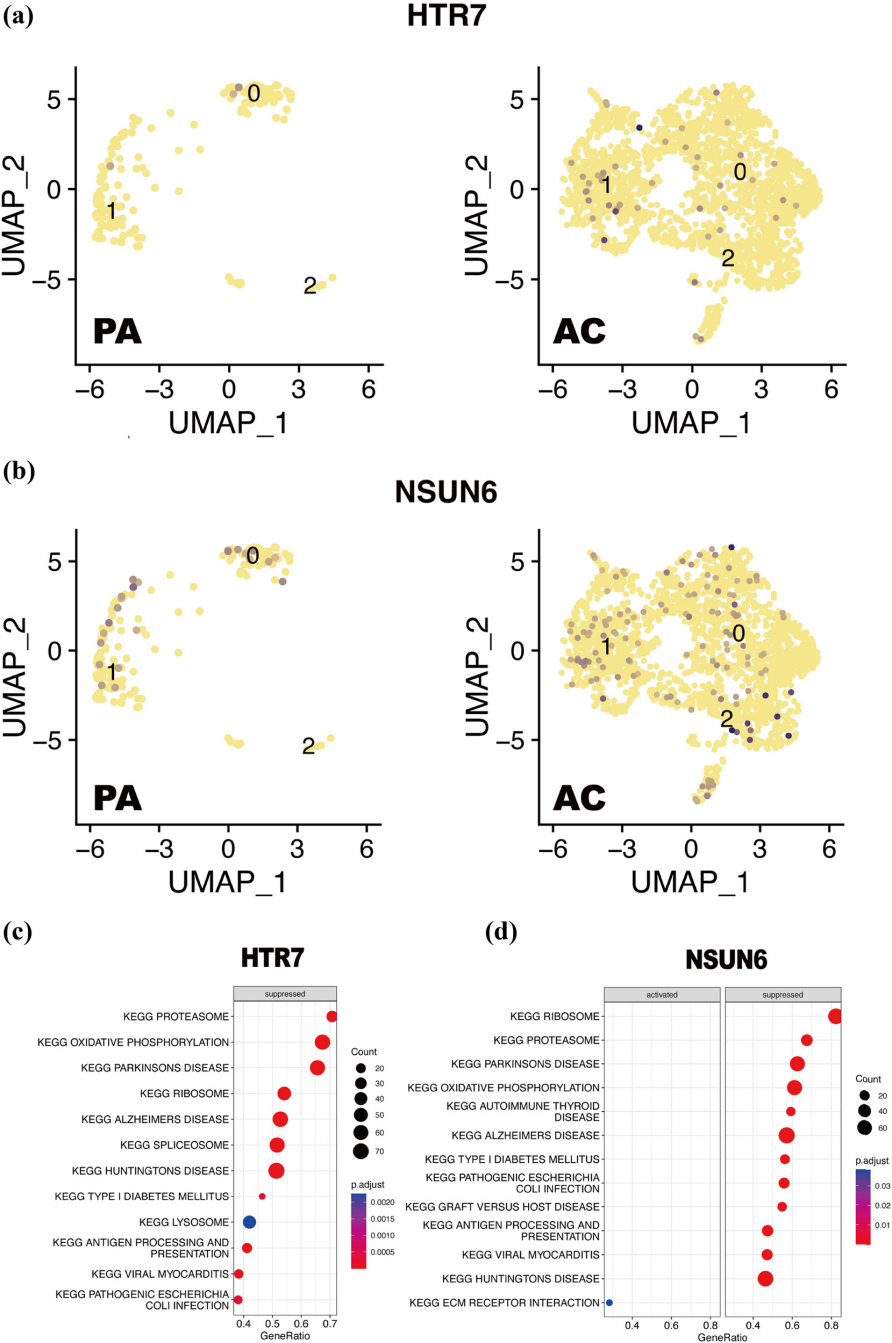
Cellular localization and GSEA of NSUN6 and HTR7. (a) UMAP shows the difference of cellular localization of HTR7 in AC and PA groups. (b) UMAP shows the difference of cellular localization of NSUN6 in AC and PA groups. (c) GSEA of HTR7. (d) GSEA of NSUN6. AC: atherosclerotic core; PA: proximal adjacent portions of carotid artery.

To further investigate the relationship of macrophages with NSUN6 and HTR7, the expression level of NSUN6 and HTR7 in different macrophage subpopulations was examined and visualized by UMAP. Compared with the PA group, both HTR7 (Figure 8a) and NSUN6 (Figure 8b) of the AC group had significantly higher expression in three clusters, especially in cluster 2. Additionally, based on the method of guilt by association, GSEA was conducted to reveal the possible biological pathways regulated by NSUN6 and HTR7 in the progress of carotid plaques. In the macrophages, HTR7 was enriched in a total of eight suppressed pathways, and the specific outcome is shown in Figure 8c. NSUN6 was enriched in a total of 15 pathways, where 12 of them were suppressed, while the others were activated. These 12 suppressed pathways mainly include protein export, proteasome, ribosome, etc. These three activated pathways include the phosphatidylinositol signaling system, ERBB signal pathway, and ECM receptor interaction (Figure 8d).

### 3.9 Pro-inflammatory factors decreased in NSUN6-overexpressed or HTR7-knocked down macrophages

**Figure 9:**
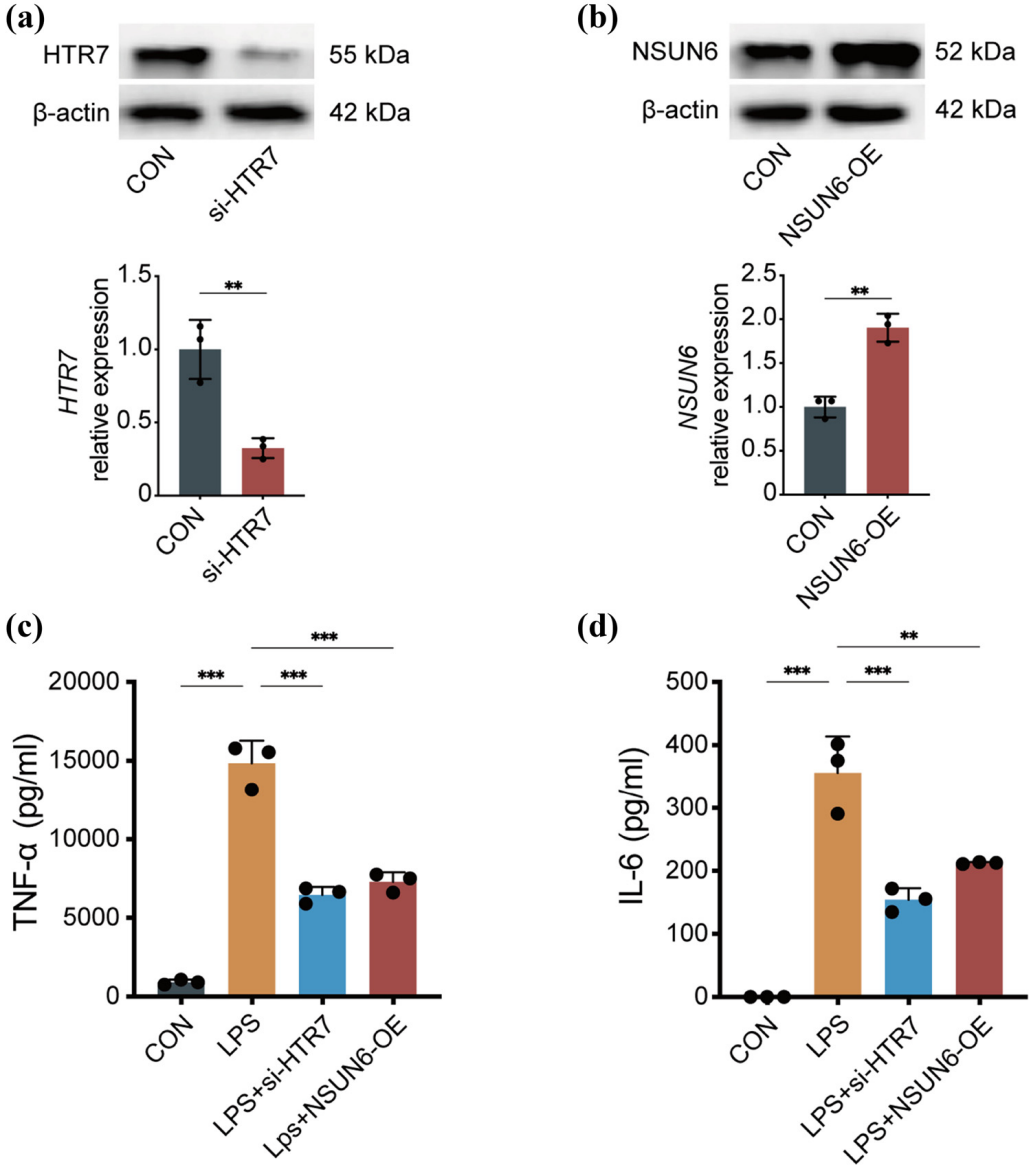
Pro-inflammatory factors decreased in NSUN6-overexpressed or HTR7-knocked down macrophages. The relative expression level of HTR7 (a) and NSUN6 (b). Level of TNF-α (c) and IL-6 (d). **P < 0.01; ***P < 0.001.

To explore the effect of NSUN6 and HTR7 on the inflammatory response of macrophages, we modulated their expression levels by either down-regulating HTR7 (Figure 9a) or up-regulating NSUN6 (Figure 9b) in macrophages. Following this, we measured the levels of TNF-α and IL-6 in response to LPS stimulation. According to the results of ELISA, TNF-α and IL-6 were lower in macrophages that overexpressed NSUN6 or had HTR7 knocked down compared to the control group (TNF-α: LPS + si-HTR7 vs LPS, *P* < 0.001; LPS + NSUN6-OE vs LPS, *P* < 0.001; IL-6: LPS + si-HTR7 vs LPS, *P* < 0.001, Figure 9c; LPS + NSUN6-OE vs LPS, *P* < 0.01, Figure 9d).

## 4 Discussion

Carotid atherosclerotic plaques exhibit a long-term course of disease [23]. Although the precise process implicated in the change of plaque vulnerability is unknown, it is widely believed that the immune system serves a vital function in the advancement of this disease [24]. Therefore, clarifying the mechanism of plaque vulnerability is significant for the treatment of atherothrombotic stroke.

In the present study, 31 unstable plaque samples and 20 stable plaque samples were selected from four gene metrics including GSE41571, GSE111782, GSE118481, and GSE12828, and 338 DEGs were identified between stable and unstable plaques. The identified KEGG pathways are predominantly associated with immune responses and lipid metabolism. For instance, IL-17 can activate the MAPK pathway [25] and NF-κB pathway, promoting inflammatory responses [26]; The gathering of blood-borne leukocytes at the site of injury, facilitated by adhesion molecules and chemoattractant cytokines, promotes the progression of plaques. Therefore, lipid accumulation, chronic inflammation, and the resulting plaque vulnerability are all closely related [27]. Therefore, these 338 DEGs are related to plaque vulnerability to a certain degree. Then, the hub genes were further identified with the Boruta and LASSO algorithms, and eight hub genes were revealed as an optimal combination that can distinguish the unstable plaque from stable plaque effectively. The PCA showed that the 31 unstable plaque samples could be distinguished from the 20 stable plaque samples only using this eight-gene combination. In addition, we conducted a model to access of predictive power of the eight-gene combination, and the AUC of the model was 0.98 with very high specificity and sensitivity, indicating that these eight hub genes can be potential biomarkers for unstable plaques. Therefore, we believe that these eight hub genes play a critical role. APOD is involved in lipid metabolism, and disruptions in lipid metabolism can affect the composition and stability of atherosclerotic plaques [28]. Although ASXL1 is primarily associated with hematological disorders [29], its role in regulating cell proliferation and gene expression may impact vascular smooth muscle cells and inflammatory responses, thereby influencing plaque stability. COL4A5 is a key component of the vascular basement membrane, crucial for maintaining vascular wall integrity [30]. It may play a role in the formation and stability of the fibrous cap of plaques. As a vascular regulatory receptor, HTR7 activation may influence vascular tone and inflammatory responses [31], potentially affecting plaque stability. INF2 is involved in cytoskeletal remodeling [32], and changes in the cytoskeleton could impact the function of endothelial cells and vascular smooth muscle cells, thereby influencing plaque stability. While NSUN6 is primarily involved in RNA methylation [33], its regulation of gene expression may indirectly affect vascular cell function and plaque stability. PDSS2 is related to mitochondrial function, and mitochondrial dysfunction is linked to oxidative stress, which plays a significant role in plaque instability [34]. RBBP7 regulates the cell cycle and apoptosis, potentially influencing smooth muscle cell proliferation and cell death within plaques [35], thereby affecting plaque stability. Therefore, these genes are involved in various biological pathways and mechanisms that may be related to the stability of atherosclerotic plaques. However, further research is needed to determine their specific roles in plaque stability. Subsequently, we evaluated these eight genes’ expression in patients, which aligned with the anticipated outcomes. Moreover, we conducted ROC and calculated the AUC of each hub gene to identify the up and down-regulated hub gene with the highest value of AUC, and HTR7 and NSUN6 were identified, respectively. HTR7 was revealed to be related to macrophage polarization [36], while NSUN6 was involved in tRNA C5-cytosine methylation [37]. Previous studies have not reported any association between these two genes and plaque vulnerability.

Studying immune cell infiltration can enhance our comprehension of the immune-related mechanisms underlying this disease. The results of CIBERSORT indicated that an increased fraction of macrophages and a decreased fraction of resting NK cells are the main changes during the state transition of plaques. Cells of monocyte/macrophage lineage are deeply involved in plaque progression [27], and uncovering some important molecular mechanisms [38,39]. However, previous studies have rarely paid attention to the number and contribution of other cells like NK cells. It was revealed that circulating NK cells were related to severe atherosclerosis [40]. In the present study, resting NK cells were decreased indicating that a certain level of them was necessary to maintain the stable state of plaques. Additionally, we investigated the correlation of the genes identified above with these infiltrated immune cells in plaques. The findings revealed a significant correlation between the hub genes and the distribution of immune cells in plaque samples with different states, providing supportive evidence for the hypothesis that immune cells serve a vital function in plaque development.

To further explore the cellular localization of these eight hub genes, scRNA-seq libraries of the isolated cells from AC plaque and PA portions were included. After quality control, 3,000 genes with high variability were identified. Using the highly variable genes, we performed dimensional reduction and PCA and utilized ten principal components as input to identify cell clusters.

The results of cell clustering showed that the immune cells subpopulation includes various cells. The abundance of immune cell subpopulations was different between AC and PA groups, especially for macrophages. This was similar to the distribution of macrophages in a different state of plaque, which indicated that the macrophages were deeply involved in not only the progression of plaque but also the formation of plaque. We subsequently examined the eight genes in these subpopulations of immune cells. The findings indicated that macrophages had the highest expression levels of these eight hub genes, and some genes, such as HTRT, were exclusively expressed in macrophages. Combined with previous findings of the immune infraction, we concluded that to a certain extent, the change of expression of these eight hub genes in macrophages promotes plaque transfer from a stable state to an unstable state.

To investigate the role of macrophages in this disease, we conducted second-level clustering of macrophages. The results showed that three clusters of macrophages were assigned. KEGG analysis indicated that cluster 0 and cluster 2 were related to the pro-inflammatory pathways and lipid metabolism, respectively, which was consistent with the results of KEGG analysis of 338 DEGs between unstable plaque and stable plaque mentioned above. Additionally, foam cells derived from macrophages are considered to be the primary factor associated with the pathogenesis of atherosclerosis in atherosclerotic plaques [41]. These results indicated that macrophages of cluster 0 have more potential to be activated into foam cells.

As HTR7 and NSUN6 were the up and down-regulated hub genes with the highest value of AUC, respectively, we further clarified their cellular localization and possible biological pathways regulated by them. Compared with PA groups, NSUN6 and HTR7 of the AC group were up-regulated in three clusters of macrophages. This change of gene expression was consistent with that in a different state of plaques that we uncovered above. Additionally, GSEA showed that NSUN6 mainly enriched in protein export, proteasome, ERBB signal pathway, oxidative phosphorylation, and the phosphatidylinositol signaling system, while HTR7 mainly enriched in the proteasome, oxidative phosphorylation, ribosome, and selenoamino acid metabolism. All of these KEGG pathways are related to cell functional states, so we speculated that NSUN6 and HTR7 affect the cell function of macrophages. When macrophages fail to effectively counteract inflammation, atherosclerotic plaques that were previously stable can progress and become unstable [7]. This hypothesis has also been verified *in vitro* experiments. We modulated the expression levels of NSUN6 and HTR7 in macrophages, followed by measuring TNF-α and IL-6 after LPS stimulation. The results indicated that HTR7 and NSUN6 do affect the inflammatory response of macrophages.

The current study revealed eight hub genes that exhibited a significant association with carotid atherosclerotic plaques. These eight hub genes might promote the progression of plaques by affecting the immune responses and lipid metabolism of macrophages. We also clarified the specific cellular localization and involved biological pathways of two genes (NSUN6 and HTR7) with high distinguishing ability, which offered insights into the molecular mechanism underlying plaque progression. This study is the first to explore the determinant genes and their cellular localizations by which carotid atherosclerotic plaques progress from stable to unstable using a combination of data from next-generation sequencing and scRNA sequencing. This study not only provides potential biomarkers for cerebrovascular events triggered by unstable plaques but also provides directions for investigating the molecular mechanisms of plaque progression. Nonetheless, the current study is limited by the absence of extensive biological research and validation using a substantial sample size. To confirm the diagnostic potential of this gene combination for unstable plaques, additional investigations employing clinical samples are necessary prior to its clinical implementation. The research focused on how these eight genes affect macrophages, and research based on animal models is required to further clarify the role of these eight genes, especially NSUN6 and HTR7 in atherosclerotic plaque progression, which may provide a new therapeutic strategy for this disease.

## 5 Conclusions

In this study, eight hub genes were identified by overlapping genes derived from Boruta and LASSO algorithms. RT-qPCR confirmed the eight hub genes’ expression in patients’ serum. We constructed a model to evaluate the predictive power of the eight-gene combination in plaque progression, and the AUC of the model was 0.98 with very high specificity and sensitivity. We then found that these eight hub genes mainly affect the immune responses and lipid metabolism of macrophages after analyzing scRNA-seq data. Thus, this study not only provides potential biomarkers for evaluating the vulnerability of plaques but also provides a new perspective for therapy targets of carotid atherosclerotic plaques.

## Abbreviations

AC: atherosclerotic core
GSEA: gene set enrichment analysis
KEGG: Kyoto Encyclopedia of Genes and Genomes
PA: proximal adjacent
PCA: principal component analysis
ROC: receiver operating characteristic curve

## Supplementary Material

Supplementary Figure
