# Supplementary material for: NSUN6 and HTR7 disturbed the stability of carotid atherosclerotic plaques by regulating the immune responses of macrophages
Source: Open Med (Wars). 2024 Oct 24;19(1):20241072. doi: 10.1515/med-2024-1072 (PMC11500533; doi:10.1515/med-2024-1072)
Supplement: Supplementary Figure [file med-2024-1072-sm.pdf]

Supplementary material

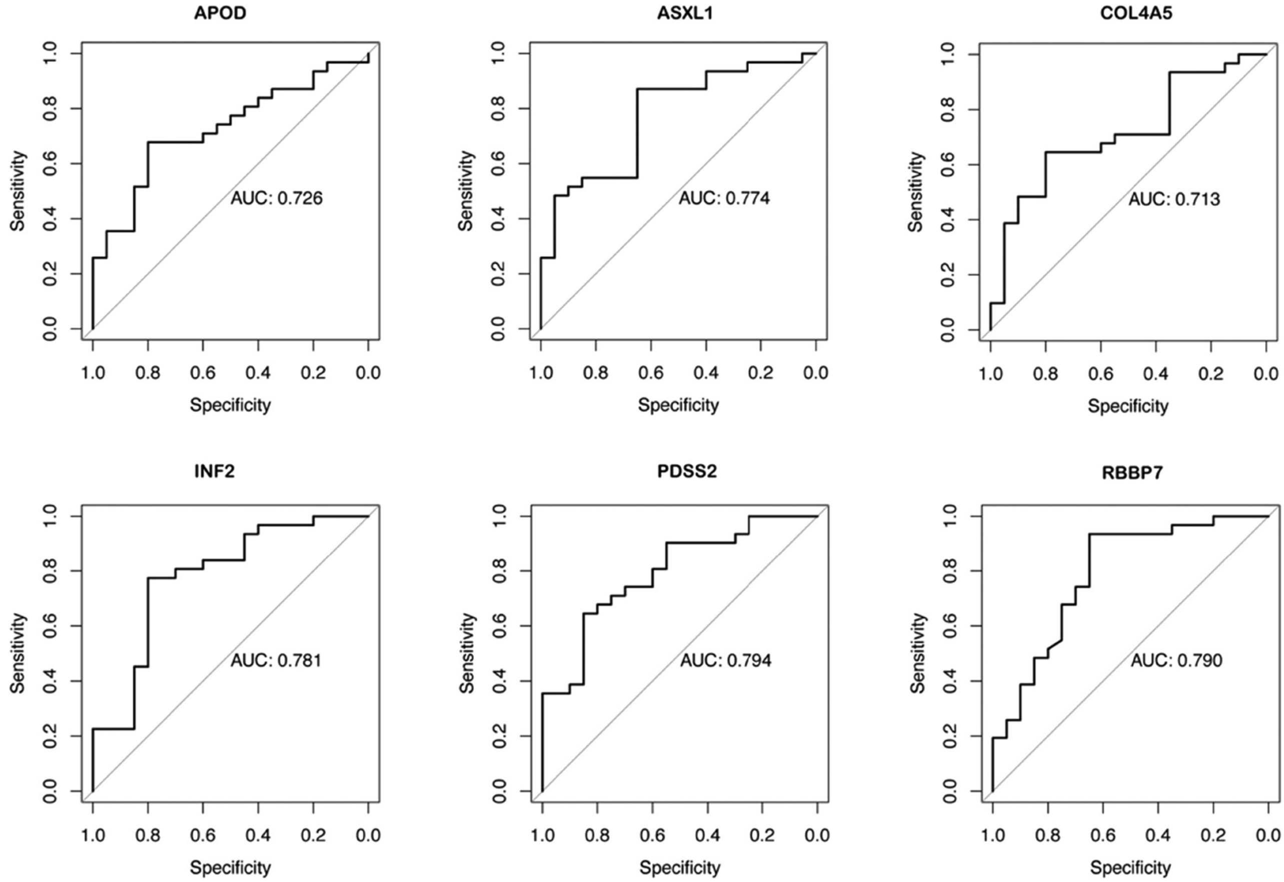

Figure S1: ROC of hub genes in distinguishing stable and unstable carotid atherosclerotic plaques.

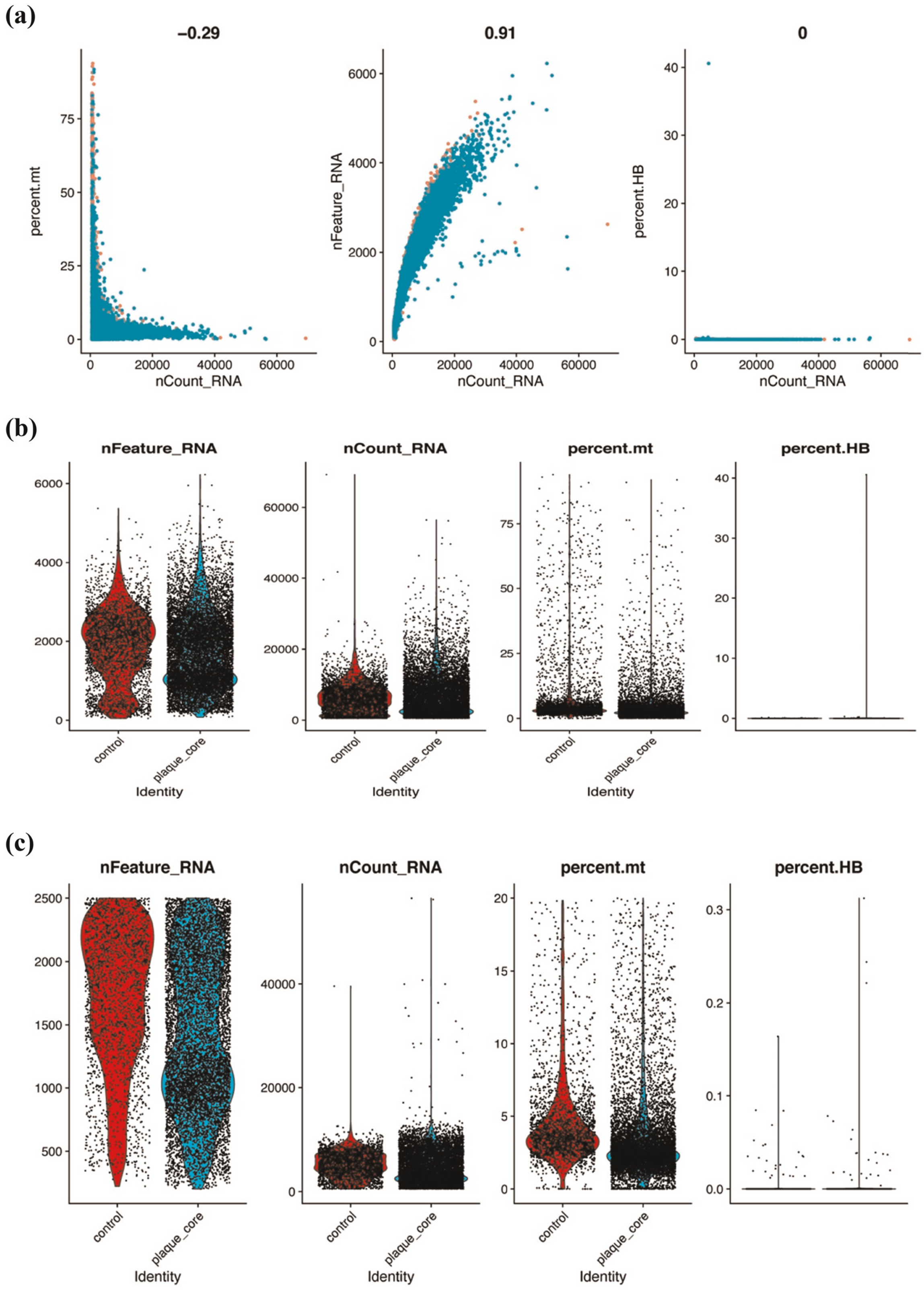

Figure S2: (a) and (b) Single-cell RNA-seq data before quality control. (c) Single-cell RNA-seq data after quality control.
